# Characterization of *Plasmodium ovale* spp. imported from Africa to Henan Province, China

**DOI:** 10.1038/s41598-019-38629-0

**Published:** 2019-02-18

**Authors:** Ruimin Zhou, Suhua Li, Yuling Zhao, Chengyun Yang, Ying Liu, Dan Qian, Hao Wang, Deling Lu, Hongwei Zhang

**Affiliations:** Department of Parasite Disease Control and Prevention, Henan Province Center for Disease Control and Prevention, Zhengzhou, 450016 P. R. China

## Abstract

As indigenous malaria has decreased over recent decades, the increasing number of imported malaria cases has provided a new challenge for China. The proportion of imported cases due to *Plasmodium ovale* has increased during this time, and the difference between *P. ovale curtisi* and *P. ovale wallikeri* is of importance. To better understand *P. ovale* epidemiology and the differences between the two subspecies, information on imported malaria in Henan Province was collected during 2010–2017. We carried out a descriptive study to analyze the prevalence, proportion, distribution, and origin of *P. o. curtisi* and *P. o. wallikeri*. It showed that imported *P. ovale* spp. accounts for a large proportion of total malaria cases in Henan Province, even more than that of *P. vivax*. This suggests that the proportion of *P. ovale* cases is underestimated in Africa. Among these cases, the latency period of *P. o. curtisi* was significantly longer than that of *P. o. wallikeri*. More attention should be paid to imported ovale malaria to avoid the reintroduction of these two subspecies into China.

## Introduction

Malaria, one of the most important global public health problems, is still a serious cause of mortality, especially in Africa. The World Health Organization (WHO) states that about 216 million cases of malaria occurred worldwide in 2016 compared with 211 million cases in 2015, and estimated that 445,000 deaths occurred from malaria globally in 2016 compared to 446,000 in 2015^1,2^. An estimated 90% of malaria cases and 92% of all malaria deaths in 2016 were in Africa^1,2^. *Plasmodium falciparum*, *P. vivax, P. ovale*, and *P. malariae* are the four species that normally cause illness in humans. Mixed infections occur in areas where more than one species is epidemic^3^. *P*. *knowlesi* is considered as a fifth pathogenic species, which is mainly a simian malaria occurring in Southeast Asia, and humans can be infected occasionally^4–6^. According to the World Malaria Report 2017^2^, *P. falciparum* is the most prevalent malarial species in Africa, accounting for 99% of estimated malaria cases in 2016. Due to this, almost all of the attention is paid to *P. falciparum*, while the other *Plasmodium* species are typically neglected.

The National Malaria Elimination Action Plan for 2010–2020 was issued in China in 2010, with the goal of achieving complete elimination by 2020^7^. There have been no local malaria cases reported in Henan Province since 2012^8^, and zero indigenous malaria cases were reported in China in 2017^9^. However, the number of imported malaria cases from overseas increased gradually, presenting a new challenge for China^10,11^. Among the imported malaria cases, all of the human malarial species have been detected, with *P. falciparum* being the main species. The proportion of *P. ovale* reported has increased and overtaken that of *P. vivax*^11^. Occasionally, malaria cases caused by *P. knowlesi* are reported in China^12^.

As one of the four main human malarial species, *P. ovale* was the last one to be reported in 1922^13^. Due to its low parasitemia and low prevalence in limited areas^14–16^, and its similar morphology with *P. vivax* and mixed infections with other *Plasmodium* species, *P. ovale* has been given relatively little attention compared with the other species, and its prevalence has apparently been underestimated^17–20^. However, by using molecular assays, it has been found that *P. ovale* occurs in most of Africa, India, and Southeast Asia^21–24^, and its prevalence has reached as high as 15% in Papua New Guinea^25^ and rural Nigeria^26^. Furthermore, according to sequence analysis, *P. ovale* is considered to be comprised of two different subspecies^21–24,27^, which were named classic and variant *P. ovale*^22^ and later formally named *P. ovale curtisi* and *P. ovale wallikeri*^23^. So far, only a few clinical, epidemiological, and therapeutic studies have reported specific data for *P. ovale* subspecies. However, the geographical distribution of *P. ovale* seems larger than previously thought based on molecular analysis.

Currently, detailed information about the incidence and distribution of malaria caused by the four *Plasmodium* species in the world is limited. Especially in epidemic regions of Africa, social and economic factors, medical facilities, and personnel capabilities might affect the identification of *Plasmodium* species, so it is more difficult to discriminate *P. o. curtisi* and *P. o. wallikeri*. Current knowledge of *P. ovale* malaria is mostly based on small trials with minor impact, case reports, and clinical observations, and there is a lack of systematic research. To date, information about the differences between *P. o. curtisi* and *P. o. wallikeri* is relatively scant, and more distinguishing features of the two subspecies should be uncovered. Presently, *P. falciparum* as the main imported *Plasmodium* species has attracted much more attention in China and the rest of the world. Thus, there have been relatively few studies about imported *P. ovale* cases in China. In the present study, based on imported malaria cases in Henan Province during 2010–2017, information was collected and the differences between *P. o. curtisi* and *P. o. wallikeri* were described and analyzed.

## Results

### Prevalence of imported malaria in Henan Province, 2010–2017

**Figure 1:**
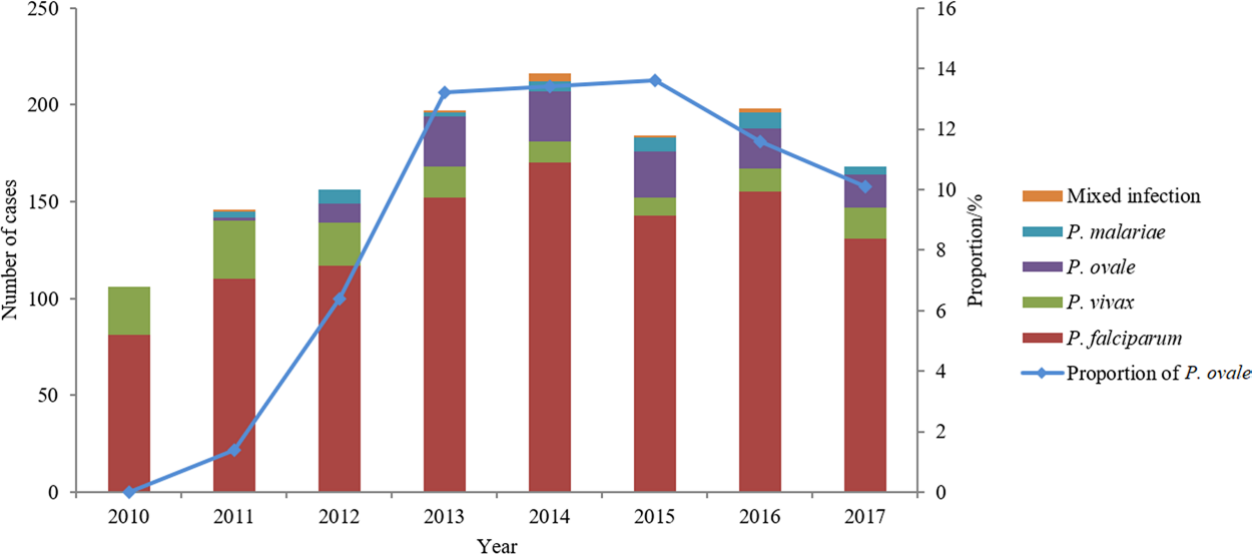
Imorted malaria cases in Henan Province, 2010–1017.

A total of 1,372 confirmed imported malaria cases were reported in Henan Province during 2010 to 2017, and no indigenous malaria case has been reported since 2012. The number of imported malaria cases increased yearly and reached a peak in 2014 (n = 216). Among all of the cases, 77.2% were infected with *P. falciparum* (n = 1059), followed by *P. vivax* (n = 142), *P. ovale* (n = 126), and *P. malariae* (n = 36), accounting for 10.3%, 9.2%, and 2.6%, respectively. One patient was infected with *P. knowlesi* imported from Indonesia in 2017. Nine patients with mixed infections accounted for 0.7% of cases, mostly with the two species *P. falciparum* and *P. ovale*. There were four cases of *P. falciparum* coinfected with *P. ovale*, two of *P. malariae* with *P. ovale*, two of *P. falciparum* with *P. vivax*, and one of *P. falciparum* with *P. malariae*. Since 2013, the number of ovale malaria cases was more than that of *P. vivax* each year, and *P. falciparum* and *P. ovale* were the two main species of imported malaria in Henan Province (Fig. 1).

### Confirmation of ovale malaria and identification of subspecies

**Figure 2:**
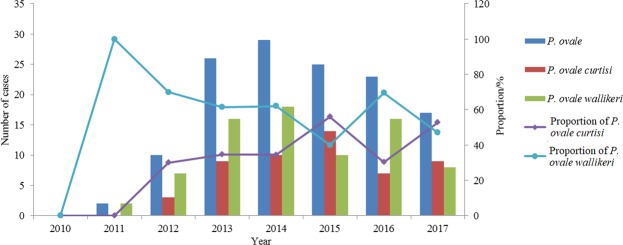
Imported malaria cases of *P. ovale* in Henan Province, 2010–2017.

Including the 6 mixed infection cases of *P. ovale* with other species, there were a total of 132 ovale malaria cases. Three patients were diagnosed by microscopy without obtaining blood samples, and the other cases were all diagnosed by microscopy and nested PCR. No case of *P. ovale* was found in 2010, but after that, the number of *P. ovale* cases increased yearly and reached a peak in 2014 (n = 29). Fifty-two (52/132, 39.4%) cases were confirmed as *P. o. curtisi* and 77 were *P. o. wallikeri* (77/132, 58.3%); the other 3 cases could not be identified and are considered as *P. ovale* spp. Except in 2015 and 2017, the number of *P. o. wallikeri* cases was much more than that of *P. o. curtisi* (Fig. 2). As for the 6 mixed infection cases, 5 cases were *P. o. wallikeri* while the other one was *P. o. curtisi*.

### Origin of the imported ovale malaria

**Figure 3:**
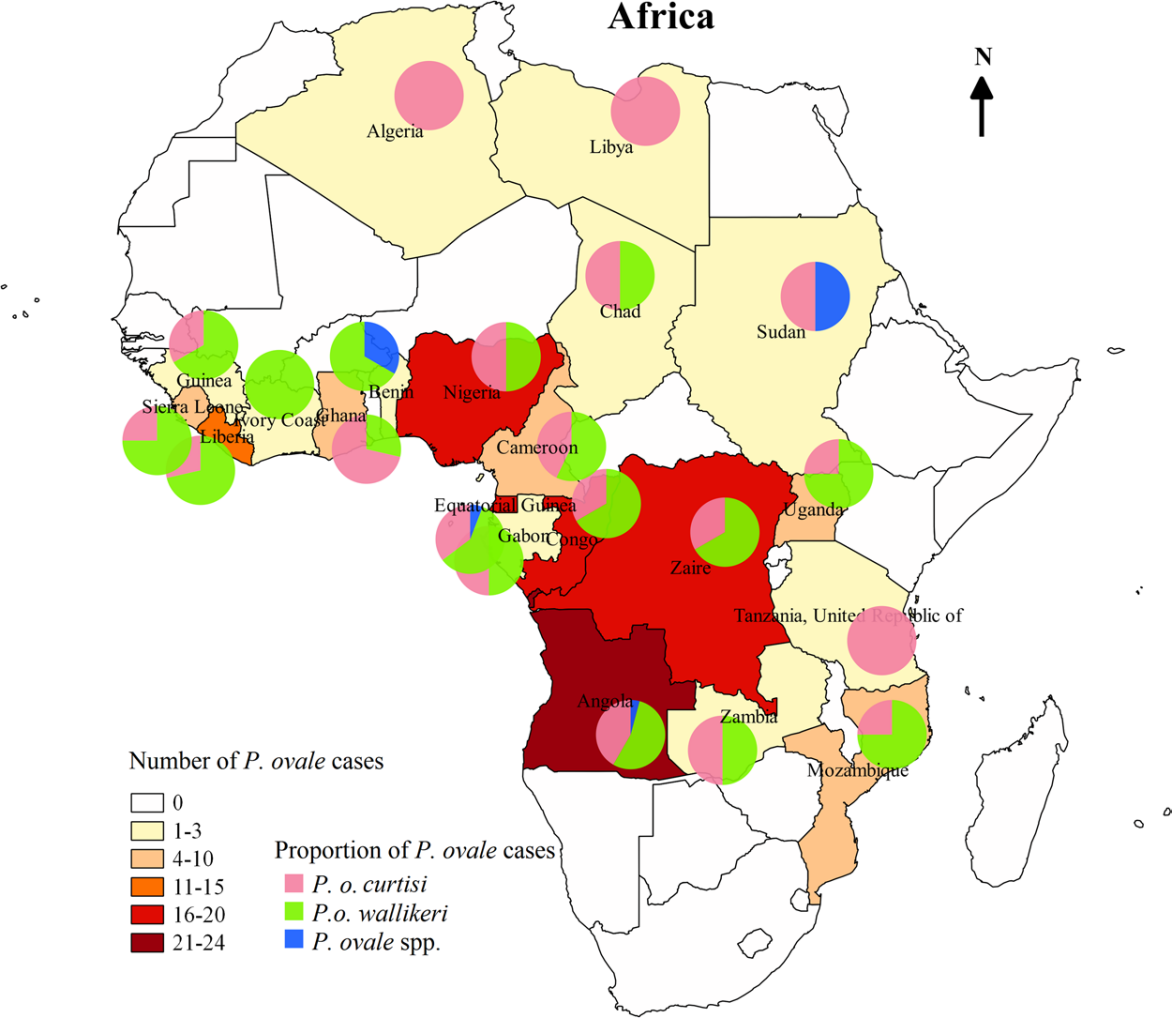
Geographic distribution of the origin countries of the imported *P. ovale* cases in Africa. The proportion of the *P. ovale* subspecies in the origin country was showed with the pie charts.

All 132 of the patients infected with *P. ovale* had the parasite imported from 20 countries in Africa, with the five top 5 countries being Angola, Congo, Equatorial Guinea, Nigeria, and Liberia, which accounted for 18.2%, 13.6%, 12.9%, 12.1%, and 10.6% of the cases, respectively. The 52 cases of *P. o. curtisi* were from 17 countries in Africa, with the top 5 countries being Angola (19.2%), Nigeria (15.4%), Congo (11.5%), Equatorial Guinea (11.5%), and Ghana (9.6%). The 77 cases of *P. o. wallikeri* were from 17 different countries in Africa, with the top 5 countries being Angola (16.9%), Congo (15.6%), Equatorial Guinea (13.0%), Liberia (13.0%), and Nigeria (10.4%). Except for the 5 countries with one case of *P. ovale*, the case numbers of *P. o. curtisi* and *P. o. wallikeri* were the same in Nigeria, Gabon, Chad, and Zambia, and the number of *P. o. curtisi* cases was more than that of *P. o. wallikeri* only in Ghana; in contrast, the number of *P. o. curtisi* cases was less than that of *P. o. wallikeri* in the other 10 countries. For the 6 mixed infection cases of *P. ovale*, the 4 cases coinfected with *P. falciparum* were from Angola (n = 2), Equatorial Guinea (n = 1), and Cameroon (n = 1), and two cases coinfected with *P. malariae* were from Congo and Liberia (Fig. 3).

### Proportion of imported malaria in the country of origin

**Figure 4:**
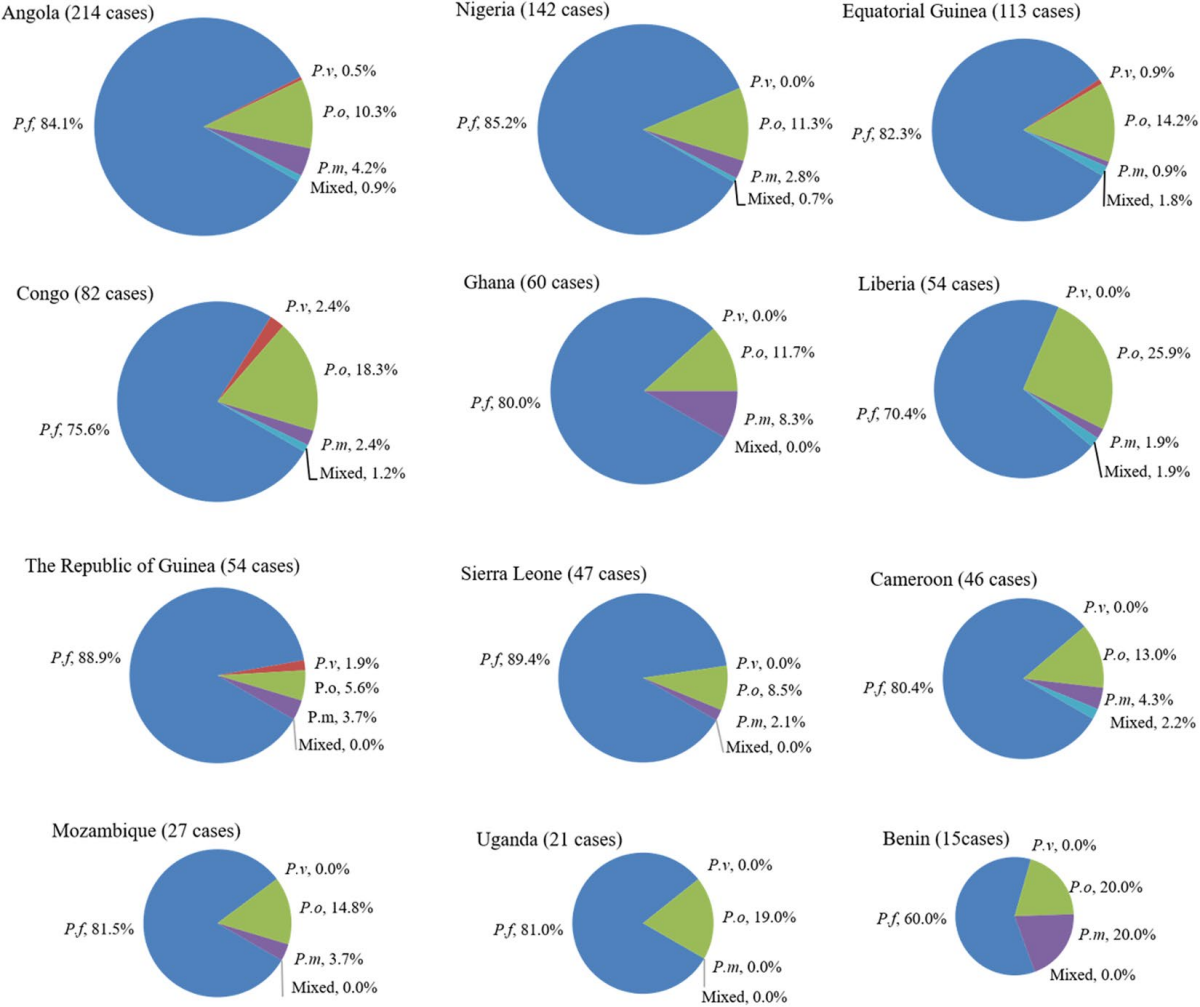
Proportion of the *Plasmodium* species among the imported malaria cases in the origin countries. The top 12 countries with most *P. ovale* cases were analyzed. *P. f*: *Plasmodium falciparum*, *P. v*: *Plasmodium vivax*, *P. o*: *Plasmodium ovale*, *P. m*: *Plasmodium malariae*, mixed: mixed infection.

For the origin countries, the total number of imported malaria cases was different, so more cases of *P. ovale* malaria did not mean a higher proportion among the four *Plasmodium* species. To obtain the proportion of the 4 *Plasmodium* species in the origin countries, the top 12 countries with the most *P. ovale* malaria cases were analyzed, and among them Liberia had the highest proportion of *P. ovale* malaria, and the Republic of Guinea had the lowest. For all 12 of the countries, except for mixed infection, the proportion of *P. falciparum* malaria cases was largest, followed by *P. ovale* and *P. malariae*, and that of *P. vivax* was smallest or equal to *P. malariae*. Except for Uganda, imported *P. malariae* existed in the other 11 countries, but imported *P. vivax* was just found in Angola, Equatorial Guinea, Congo, and Guinea (Fig. 4).

### Temporal distribution of ovale malaria

**Figure 5:**
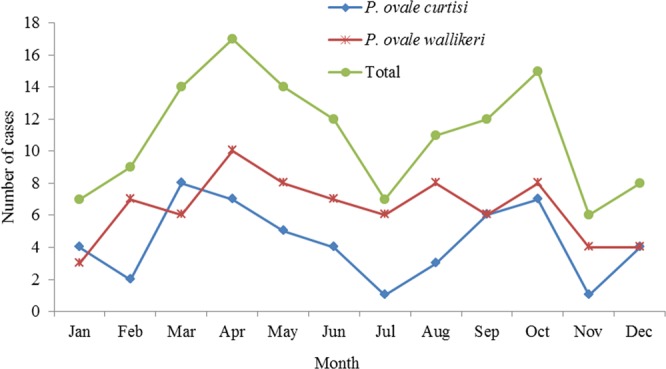
Monthly distribution of reported *P. ovale* cases, 2010–2017.

There were ovale malaria cases reported every month, but the peaks appeared in April and October for all of the cases, accounting for 12.9% (n = 17) and 11.4% (n = 15), respectively. Compared with the other months, cases in January, July, and November were fewer. For *P. o. curtisi*, two peaks appeared in March and October, and cases in February, July, and November were fewer compared with the other months. For *P. o. wallikeri*, most cases were concentrated in February to October without an obvious peak, accounting for 85.7% (66/77) of the total (Fig. 5).

### Interval between arrival in China and illness onset

**Figure 6:**
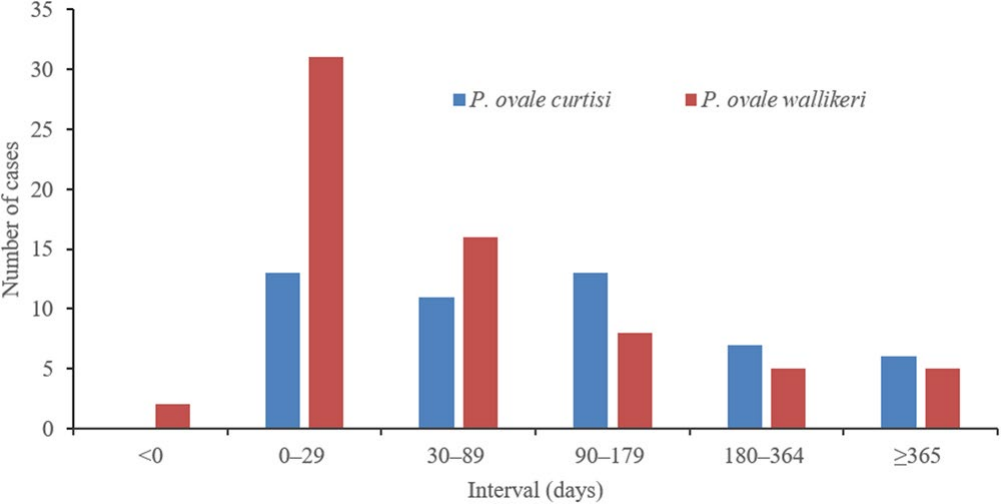
The interval between date of arrival in China and illness onset.

Except for the 6 mixed infection cases of *P. ovale* with other species, among the 126 imported ovale malaria cases, 120 (95.2%) had complete information about the date of arrival in China and illness onset, from which the interval days could be calculated. For the interval between arrival and disease onset, the shortest was 23 days before returning to China, the longest was 1265 days after arrival in China, the median was 59 days, and the interquartile range was (14–160) days. Three (2.5%) patients had symptoms onset before arrival in China. After arriving in China, 45 (37.5%) patients had symptoms onset within one month. The median and interquartile range of *P. o. curtisi* was 97.5 days (7.5–188.5), and that of *P. o. wallikeri* was 31 days (14–99). The difference between *P. o. curtisi* and *P. o. wallikeri* for the interval of arrival and disease onset was statistically significant (*Z* = 10.159, *p* = 0.001). More patients infected with *P. o. wallikeri* (33/67, 49.3%) had illness onset within one month after arrival in China compared with people infected with *P. o. curtisi* (13/50, 26.0%), and this difference was statistically significant (Pearson χ^2^ = 5.013, *p* = 0.010). In contrast, fewer patients infected with *P. o. wallikeri* (18/67, 26.7%) had symptoms onset after returning three months later compared with those with *P. o. curtisi* (26/50, 52.0%), and the difference was statistically significant (Pearson χ^2^ = 7.709, *p* = 0.005) (Fig. 6).

### Interval between disease onset and diagnosis

Except for the 6 mixed infection cases of *P. ovale* with other species, information about the date of symptom onset and diagnosis was collected from the 124 imported ovale malaria cases, and this allowed for calculating the interval days. For the interval between disease onset and diagnosis, the shortest was 1 day, the longest was 59 days, the mode was 1 day, and the median and interquartile range was 4 days (2–7). In addition, 49.2% (61/124) of the patients were diagnosed within 3 days after disease onset, and 78.2% (97/124) were diagnosed within one week. However, there were a few patients diagnosed 15 days later, or even 1 month after disease onset, accounting for 6.5% (8/124) and 2.4% (3/124) of cases, respectively. The differences between *P. o. curtisi* and *P. o. wallikeri* for the interval of disease onset and diagnosis were not statistically significant for any groups.

## Discussion

After the launch of the National Malaria Elimination Action Plan for 2010–2020 in China in 2010^7^, local malaria cases have been reduced yearly until no indigenous malaria case has been reported in Henan Province since 2012^8^. However, reported malaria cases have increased every year and reached a peak in 2014, but decreased by about 15% in 2015, which might be affected by reducing travel due to the transmission of Ebola in western Africa during 2014–2015; however, the proportion of *P. ovale* cases was increased in 2015. At present, the few studies are available about imported malaria in China that shows Africa is the top area of origin, and Angola, Congo, Equatorial Guinea, Ghana, and Nigeria are the most common countries of origin^10,11,28,29^. However, there are relatively few studies about imported malaria that describe the proportion of the four human *Plasmodium* species in Chinese patients following African travel. Imported *P. falciparum* cases accounted for 83.4% of the total in Jiangsu Province during 2011–2014, although the proportion of *P. ovale* cases increased yearly to 3.7%, 9.6%, 8.8%, and 13.0%, respectively^28^. In the present study, the proportion of imported *P. falciparum* cases was 77.2% during 2010–2011 in Henan Province, which remained about 75.0% in these years, while imported *P. ovale* cases accounted for 0.0%, 1.4%, 6.4%, 13.2%, 12.0%, 13.0%, 10.6%, and 10.1%, respectively, which was similar to the results in Jiangsu Province^28^.

In this study, we found that *P. ovale* cases were imported from 20 countries in Africa, with Angola, Congo, Equatorial Guinea, Nigeria, and Liberia being the 5 top origin countries for the 132 imported *P. ovale* cases. *P. ovale* is widespread in Africa and fairly common. However, studies about the prevalence of *P. ovale* in Africa are relatively few. Until recently, species-level diagnosis of malaria has been difficult in some African countries, so the prevalence of ovale malaria may be underestimated. Also, differences in prevalence may be due to different characteristics of Chinese travelers compared to locals in African countries. In addition, factors, such as length of exposure, access to care, past treatment, usage of prophylaxis, and clinical presentation, could explain these differences, which need more study. There are some limitations to this study: the results were obtained without carefully planned sampling, so the largest number of imported malaria cases just showed which origin country was the most common in Henan Province, and the true prevalence of *Plasmodium* species in the origin country cannot be estimated from this proportion. The proportion of *P. ovale* malaria in cases travelling to Liberia was highest (25.9%), while that of Angola was just 10.3%. However, there were only 20 African countries analyzed in this study, which were the most common sites of origin with *P. ovale* in Henan Province, so more surveys about the prevalence of *P. ovale* in Africa should be performed.

Since *P. ovale* was classified into two different subspecies, *P. o. curtisi* and *P. o. wallikeri*^23^, more attention has been paid to the detection and genotype of the two subspecies^30–35^, and studies focusing on the prevalence and distribution of *P. ovale* spp. have been relatively few. Among 98 imported cases of *P. ovale* in Jiangsu Province, China, the proportions of *P. o. curtisi* and *P. o. wallikeri* were 48% and 52%, which are comparable^12^. In this study, *P. o. curtisi* and *P. o. wallikeri* accounted for 39.4% and 58.3%, respectively, with the number of *P. o. curtisi* cases being less than that of *P. o. wallikeri* in most countries. The proportion of the two subspecies is similar to that in Ethiopia and Senegal. In northwest Ethiopia, out of 9 *P. ovale* cases, 7 patients were infected with *P. o. wallikeri* while the other 2 were infected with *P. o. curtisi*^36^. In Senegal, out of 235 malaria cases, 4 patients were infected with *P. ovale*, 3 with *P. o. wallikeri*, and 1 with *P. o. curtisi*^37^.

For indigenous malaria, the peak has appeared around August and September in China. However, there is no obvious peak for imported malaria^8^. In this study, the peaks of total *P. ovale* and *P. o. curtisi* were similar and appeared in March and October, which might be related with the Chinese festivals, the Spring Festival in February, and National Day in October. Migrant workers prefer to return to China during these festivals. However, no obvious peak was observed for *P. o. wallikeri*. The differences in temporal pattern between *P. o. curtisi* and *P. o. walliker* deserve more study.

It was difficult to obtain the exact date of malaria infection in this epidemiological investigation. However, imported malaria cases are suitable for observing the latency period of malaria. The date of arrival in China was used as the date of malaria infection, and the intervals between the date of arrival and illness onset were calculated. Although imprecise, this can be used as a proxy to estimate the latency period, for which *P. ovale* malaria is mostly observed through imported cases^28,38^. Nolder *et al*. observed that the latency period of *P. o. curtisi* (85.7 days) was much longer than that of *P. o. wallikeri* (40.6 days)^38^, whereas the latency period of the two subspecies showed no difference in the study of Cao *et al*.^28^. In the present study, the latency period of *P. o. curtisi* (97.5 days) was more than three times as long as that of *P. o. wallikeri* (31 days), which is similar to the work of Nolder *et al*.^38^. The longest latency period in this study was 1265 days, which happened in a case of *P. o. curtisi*; similarly, the longest was also a case of *P. o. curtisi* in the study of Nolder *et al*. (1083 days)^38^. The liver stage of malaria commonly exists for parasites of *P. ovale* and *P. vivax*; the hypnozoites in the liver last months or years after infection, which results in a long latency period. This makes it quite difficult to diagnose imported malaria cases in nonendemic countries because the presenting illness may be unrelated to recent travel abroad^39–41^. More studies about the latency period of *P. ovale* and *P. vivax* parasites will improve the diagnosis of imported malaria.

Henan Province, located in the Middle East of China, has a warm temperate zone, subtropical, humid-semi-humid monsoon climate. The climate and environment are suitable for the breeding of *Anopheles*, including *Anopheles sinensis* and *Anopheles anthropophagus*, so the threat of malaria reintroduction remains. Currently, for imported malaria, timely diagnosis, standardized treatment, and preventing further transmission are the key strategies to eliminate sources of infection in China.

## Conclusion

This study illustrates that imported *P. ovale* cases account for a large proportion of the total cases in Henan Province, even larger than *P. vivax* cases. The proportion of *P. ovale* cases is underestimated in the origin countries of Africa, which is large and exceeded only by *P. falciparum*. We confirmed that the latency period of *P. o. curtisi* is much longer than that of *P. o. wallikeri*. It is recommended that more attention be paid to imported *P. ovale* cases to avoid reintroduction in China.

## Methods

### Data collection

Information on imported malaria in Henan Province was collected from case investigation reports and the Disease Surveillance Information Report Management system of the China Centers for Disease Control and Prevention (CCDC), including gender, age, occupation, travel history, date of arrival in China, date of symptom onset, date of diagnosis, and reported institution.

### Species and subspecies confirmation

All of the cases were initially diagnosed by blood smear microscopy and/or rapid diagnostic tests (RDT) at the basic level of the hospital or CDC, then all of them were confirmed by the Henan Provincial Reference Laboratory for Malaria Diagnosis using nested polymerase chain reaction (PCR) and/or blood smear microscopy. The microscopy was performed by microscopists through the examination of thick and thin blood film at 1000×magnification. Rapid diagnostic tests were used to detect malaria antigens following the manufacturer’s instructions by laboratory staff. Nested PCR was performed using the methods published^42,43^. Subspecies of *P. ovale* were confirmed by nested PCR following sequencing according to previously published methods^23,30^.

### Data analysis

All of the case information was collected and input into SSPS v.21.0 (Statistical Product and Service Solutions), in which all of the calculations and analyses were performed. The differences were compared by Wilcoxon rank-sum test, Chi-square, or Fisher’s exact tests, and a two-sided *p* value of <0.05 was considered statistically significant. Geographic distribution was conducted by geographic information system (GIS)-based spatial analysis, which was fulfilled by QGIS (Quantum GIS) v2.18.19.

### Ethics approval and consent to participate

All of the identifiers of patients were removed from the data. Informed consent was obtained from all of the patients. This study was approved by the Ethical Committee of the Henan Province Center for Disease Control and Prevention. All of the methods were performed in accordance with the relevant guidelines and regulations of the Henan Province Center for Disease Control and Prevention.

## Supplementary information

Characterization of Plasmodium ovale spp. imported from Africa to Henan Province, China

## Data Availability

The datasets used and/or analyzed during the current study are available from the first/corresponding author on reasonable request.
