# Supplementary material for: Characterization of Plasmodium ovale spp. imported from Africa to Henan Province, China
Source: Sci Rep. 2019 Feb 18;9:2191. doi: 10.1038/s41598-019-38629-0 (PMC6379410; doi:10.1038/s41598-019-38629-0)
Supplement: Supplementary file 1 — Characterization of Plasmodium ovale spp. imported from Africa to Henan Province, China [file 41598_2019_38629_MOESM1_ESM.docx]

**Characterization of *Plasmodium ovale* spp. imported from Africa to Henan Province, China**

Ruimin Zhou, Suhua Li, Yuling Zhao, Chengyun Yang, Ying Liu, Dan Qian, Hao Wang, Deling Lu, Hongwei Zhang*

Department of Parasite Disease Control and Prevention, Henan Province Center for Disease Control and Prevention, Zhengzhou, 450016, P. R. China

* Corresponding [zhwei69@163.com](mailto:zhwei69@163.com)

Table 1 Origin of imported *ovale* malaria cases in Henan Province, 2010-2017

| **Origin country** | ***P. ovale curtisi*** | ***P. ovale wallikeri*** | **Total** |
| --- | --- | --- | --- |
|  | **No. (%)** | **No. (%)** | **No. (%)** |
| Angola | 10 (19.2) | 13 (16.9) | 23+1^a^ (18.2) |
| Congo | 6 (11.5) | 12 (15.6) | 18 (13.6) |
| Equatorial Guinea | 6 (11.5) | 10 (13.0) | 16+1^a^ (12.9) |
| Nigeria | 8 (15.4) | 8 (10.4) | 16 (12.1) |
| Liberia | 4 (7.7) | 10 (13.0) | 14 (10.6) |
| Ghana | 5 (9.6) | 2 (2.6) | 7 (5.3) |
| Cameroon | 3 (5.8) | 4 (5.2) | 7 (5.3) |
| Mozambique | 1 (1.9) | 3 (3.9) | 4 (3.0) |
| Sierra Leone | 1 (1.9) | 3 (3.9) | 4 (3.0) |
| Uganda | 1 (1.9) | 3 (3.9) | 4 (3.0) |
| Benin | 0 (0.0) | 2 (2.6) | 2+1^a^ (2.3) |
| Guinea | 1 (1.9) | 2 (2.6) | 3 (2.3) |
| Gabon | 1 (1.9) | 1 (1.3) | 2 (1.5) |
| Chad | 1 (1.9) | 1 (1.3) | 2 (1.5) |
| Zambia | 1 (1.9) | 1 (1.3) | 2 (1.5) |
| Algeria | 1 (1.9) | 0 (0.0) | 1 (0.8) |
| Ivory Coas | 0 (0.0) | 1 (1.3) | 1 (0.8) |
| Libya | 1 (1.9) | 0 (0.0) | 1 (0.8) |
| Sudan | 0 (0.0) | 1 (1.3) | 1 (0.8) |
| Tanzania | 1 (1.9) | 0 (0.0) | 1 (0.8) |
| Total | 52 (100) | 77 (100) | 129+3 ^a^ (100) |

^a^ The subspecies could not be identified and considered as *P. ovale* spp.

Table 2 The interval between date of illness onset and diagnosis

| **Interval (days)** | **Total** | ***P. ovale curtisi*** | ***P. ovale wallikeri*** | ***p* value** |
| --- | --- | --- | --- | --- |
|  | **No. (%)** | **No. (%)** | **No. (%)** |  |
| 0 | 9 (7.3) | 3 (5.8) | 6 (8.0) | 0.578 |
| 1–3 | 51+1^a^ (41.9) | 22 (42.3) | 34 (45.3) | 0.853 |
| 4–7 | 36 (29.0) | 16 (30.8) | 21 (28.0) | 0.739 |
| 8–10 | 10 (8.1) | 4 (7.7) | 6 (8.0) | 0.886 |
| 11–15 | 6 (4.8) | 4 (7.7) | 2 (2.7) | 0.212 |
| 16-29 | 6+2^a^ (6.5) | 2 (3.8) | 4 (5.3) | 0.654 |
| ≥30 | 3 (2.4) | 1 (1.9) | 2 (2.7) | 0.754 |
| Total | 121+3^a,b^ (100) | 52 (100) | 75 (100) |  |

^a^ The subspecies could not be identified and considered as *P. ovale* spp.

^b^  2 patients for whom date of symptom onset is not clear are not included, 6 mixed infective cases of *P. ovale* with other species are not included.
